# Comparative Evaluation of High‐Frequency Microneedling Using a Layering Technique Versus Conventional Technique for Facial Rejuvenation

**DOI:** 10.1111/jocd.70651

**Published:** 2026-02-25

**Authors:** Ye Tang, Kejia Wu, Xin Song, Zhishang Lu, Meina Fan, Manli Qiao

**Affiliations:** ^1^ Department of Dermatology Changzhou Traditional Chinese Medicine Hospital, Tianning District Changzhou City Jiangsu Province China; ^2^ Departmeny of Dermatology Changzhou Third People's Hospital Changzhou City Jiangsu Province China

**Keywords:** clinical efficacy, facial rejuvenation, high‐frequency microneedling layering technique, patient satisfaction, skin quality

## Abstract

**Objective:**

To evaluate the effectiveness of high‐frequency microneedling (HFM) using a layering technique versus conventional HFM for facial rejuvenation, with the Fitzpatrick Wrinkle and Laxity Classification Scale (FWCS) score at 3 months post‐treatment as the primary endpoint.

**Methods:**

A prospective non‐randomized controlled study was conducted on 30 patients undergoing facial rejuvenation at Changzhou TCM Hospital (January–December 2024), who were divided into two groups (*n* = 15 each). The conventional group received standard HFM, while the layered group received HFM with needle length, power, and pulse width adjusted for superficial, middle, and deep skin layers. The primary endpoint was the Fitzpatrick Wrinkle and Laxity Classification Scale (FWCS) score at 3 months post‐treatment. Secondary endpoints included FWCS scores at 1 and 2 months, VISIA‐derived skin texture/pore scores, Global Aesthetic Improvement Scale (GAIS) scores, Cutometer skin rebound rate, and patient satisfaction. Assessments were performed pre‐treatment and at 1, 2, and 3 months post‐treatment. Statistical analyses were conducted using SPSS 22.0, with linear mixed‐effects models for repeated measures, ordinal methods for GAIS, and Fisher's exact test for satisfaction.

**Results:**

Pre‐treatment FWCS scores showed no significant difference (*p* > 0.05). At 1 month, the layering group showed a significantly greater FWCS score reduction (2.53 vs. 1.34, *p* < 0.05). FWCS scores remained significantly lower in the layering group at 2 and 3 months (*p* < 0.05). Skin texture improvement was significantly better maintained in the layering group at 2 and 3 months (*p* < 0.05). Pore improvement was significantly better in the layering group only at 3 months (*p* < 0.05). GAIS scores indicated significantly superior rejuvenation effects in the layering group (*p* < 0.001). Patient satisfaction was significantly higher in the layering group (93.33% vs. 60.00%, *p* < 0.05). No serious adverse reactions occurred; transient redness and mild discomfort resolved quickly in both groups.

**Conclusion:**

The HFM layering technique demonstrates superior efficacy over conventional HFM for facial rejuvenation, with more significant and sustained improvements in wrinkles, skin texture, pores, and elasticity, as well as higher patient satisfaction. Both techniques are safe, though the layered approach is associated with slightly longer downtime.

## 1 Introduction

Facial aging is a complex physiological process characterized by progressive volume loss, soft tissue sagging, and gradual decline in skin elasticity, with these signs interacting to form a multi‐layered degenerative pattern [1, 2]. Traditional facial rejuvenation treatments—including injectable fillers (at risk of overfilling [3]), photoelectric therapy/chemical peels (associated with skin irritation and recovery periods [4, 5]), and surgery (long downtime and high risks [6])—have limitations, driving demand for minimally invasive, effective alternatives [7].

High‐frequency microneedling (HFM), an emerging option, combines physical microneedle stimulation with radiofrequency thermal effects to trigger collagen and elastin regeneration in deep skin layers, achieving skin tightening, wrinkle reduction, and texture improvement [8, 9, 10]. Compared to traditional methods, HFM offers superior efficacy, minimal invasiveness, and shorter recovery [11], as supported by international studies [12]: highlighted its role in multi‐level anti‐aging strategies, while [13] noted its potential to enhance dermal fiber remodeling. Recent innovations in HFM include a layered technique, which addresses a key limitation of conventional HFM—single fixed parameters (e.g., uniform needle length/power) that may cause overstimulation of superficial layers or insufficient deep‐layer penetration [14, 15]. By adjusting needle length (0.7 mm for superficial, 1.4 mm for middle, 2.1 mm for deep layers), power (2–4 W/6–8 W/8–12 W), and pulse width (20–40 ms/60–80 ms/100–150 ms) for each skin layer [16], the layered technique enables precise thermal delivery, promoting multi‐depth collagen mesh remodeling and uniform energy distribution—mechanisms hypothesized to enhance efficacy [17]. Gouveia et al. [17] further confirmed that layer‐specific energy modulation improves treatment targeting, while Wang et al. [18] reported similar benefits of multi‐depth stimulation for wrinkle reduction.

This study aimed to evaluate the clinical efficacy and safety of high‐frequency microneedling using a layering technique versus conventional HFM for facial rejuvenation, with a focus on wrinkle/laxity improvement, skin quality enhancement, and patient satisfaction.

## 2 Data and Methods

### 2.1 General Information

A prospective non‐randomized controlled study was conducted at Changzhou Hospital of Traditional Chinese Medicine from January to December 2024. Thirty patients were included and assigned to the conventional treatment group or layered treatment group based on their own treatment preference (*n* = 15 each, no randomization protocol applied), with a CONSORT‐style patient flow diagram (Figure S1, detailing screening, enrollment, and completion rates).

To verify group balance, a dedicated baseline characteristics table (Table S1) was established, including demographics (age, gender), Fitzpatrick skin type, baseline outcome measures (FWCS score, VISIA texture/pore score, skin rebound rate), prior facial treatment history, and lifestyle factors (smoking status).

Key baseline data showed no significant differences between groups: the conventional group had a mean age of 38.25 ± 5.88 years (range 32–47 years) versus 37.18 ± 6.22 years (range 30–49 years) in the layered group (*p* = 0.723); baseline FWCS score, VISIA texture/pore score, and skin rebound rate were also comparable (all *p* > 0.05, detailed in Table S1 and Tables 2, 3, 4, 5). Additionally, no patients in either group had prior facial cosmetic treatments (e.g., injectables, lasers) in the past year, and smoking rates were consistent (13.33% in both groups, *p* = 1.000).

#### 2.1.1 Sample Size Calculation

The sample size was determined based on prior preliminary experimental data and a published study on high‐frequency microneedling (HFM) layered therapy. Jiang et al. [16] reported a large effect size (Cohen's *d* = 0.82) for the between‐group difference in Fitzpatrick Wrinkle and Laxity Classification Scale (FWCS) scores (the primary endpoint of this study) after HFM treatment. Using G*Power 3.1 software for calculation, we set the following parameters: two‐independent‐samples *t*‐test, two‐tailed test, α (type I error rate) = 0.05, power (1‐β, type II error rate) = 0.80, and effect size = 0.82. The results showed that the minimum sample size required per group was 12 cases. Considering a potential dropout rate of 20% (common in facial rejuvenation clinical studies [19]), we finally determined to enroll 15 cases per group, with a total of 30 cases in the study. No patients were lost to follow‐up during the study period, and all 30 cases were included in the final statistical analysis.

### 2.2 Ethics Approval

This study was conducted in strict accordance with the Declaration of Helsinki (2013 revision) and was approved by the Ethics Committee of Changzhou Hospital of Traditional Chinese Medicine (approval number: C‐2024‐003). No trial registration was required as it was a single‐center, prospective non‐randomized observational study focusing on clinical efficacy comparison of existing treatment techniques. Before enrollment, all participants were fully informed of the study purpose, treatment procedures, potential risks (e.g., transient erythema), and data usage (including clinical photographs for scientific reporting). Each participant signed a written informed consent form, which was archived in the hospital's research ethics management system for 5 years for future verification.

### 2.3 Inclusion and Exclusion Criteria

Inclusion criteria: (1) Age 30–50 years old; (2) Score of 3–6 according to the Fitzpatrick Wrinkle and Laxity Classification Scale (FWCS); (3) Possessing normal cognitive ability (to ensure understanding of consent content); (4) Not having received facial surgery or non‐surgical cosmetic treatments and having no such plans in the next year; (5) No implanted electronic devices such as pacemakers in the body; (6) Having signed the written informed consent form voluntarily (consistent with the ethics approval requirement).

Exclusion criteria: (1) history of skin tumor; (2) presence of acute inflammation of large areas of the skin; (3) presence of metal foreign objects or fillers in the treatment area, which affects the therapeutic effect; (4) suffering from serious heart, lung and other systemic diseases or infectious diseases; (5) women who are pregnant or breastfeeding; (6) presence of active bacterial, fungal, or viral infections in the treatment area of the face, or generalized/localized dermatitis, keloidal, or diagnosed with autoimmune disease; (7) subjects with central nervous system disorders or psychiatric disorders who are unable to fully understand and cooperate; (8) the presence of other complications, medications that in the sole opinion of the investigator may interfere with the evaluation of the study, or participation in the study that in the opinion of the investigator may result in a greater risk to the subject.

### 2.4 Treatment Protocol

After topical anesthesia, all patients were first subjected to standard disinfection procedures. The high‐frequency microneedling (HF microneedling) were used for treatment. Specific treatment parameters (needle length, power, pulse width) were set according to the group, as detailed in Table 1. During the treatment, the depth of microneedles was adjusted according to different parts and immediate reaction, and the overlap rate was 20%. The skin reaction was closely observed.

**TABLE 1:** Comparison of treatment parameters between two groups of high‐frequency microneedling.

Group	Subgroup	Needle length	Therapeutic power	Therapeutic pulse width
Conventional treatment group (*n* = 15)	—	1.2–2.0 mm	6–10 W	60–150 ms
Layered treatment group (*n* = 15)	Superficial layer	0.7 mm	2–4 W	20–40 ms
	Middle layer	1.4 mm	6–8 W	60–80 ms
	Deep Layer	2.1 mm	8–12 W	100–150 ms

*Note:* (1) Parameter adjustment: Basic parameters were fixed for all patients, but needle length/power were (±0.1 mm/±1 W) intra‐procedure based on facial regions (e.g., thinner skin on cheek: needle length reduced by 0.1 mm; thicker skin on forehead: power increased by 1 W) [20]. (2) Overlap rate clarification: The 20% overlap rate refers to the overlapping area between adjacent treatment passes, ensured by grid‐marking the facial area (1 cm × 1 cm grids) to avoid missing or repeated stimulation [11].

Anesthesia details: 2 mm thick 2% lidocaine cream (Manufacturer: Shanghai Harvest Pharmaceutical Co. Ltd., Approval No.: H20053710) was uniformly applied to the facial treatment area, fully covered with plastic wrap for 1 h to ensure anesthesia depth reached the superficial dermis (verified by pinprick test before treatment, no pain response indicated effective anesthesia).

Operator qualification: All treatments were performed by the same practitioner with 5 years of clinical experience in high‐frequency microneedling (certified by the Chinese Society of Aesthetic Dermatology) to avoid operator variability.

Treatment operation: Using the high‐frequency microneedling‐HF microneedling, both groups adopted 2 passes of seamless carpet‐style operation with a 20% overlap rate (ensured by marking the treatment area with a grid to avoid missing or repeated stimulation). The treatment head was kept vertical to the skin, and energy was released only when tightly fitted to prevent epidermal damage.

Post‐treatment care: Immediately after treatment, a sterile cold compress (4°C, sterile gauze wrapped) was applied for 20 min to reduce erythema; patients were instructed to apply medical repair cream (main ingredients: hyaluronic acid, ceramide) twice daily for 1 week, avoid washing the face with hot water for 3 days, and refrain from makeup for 5 days.

Prohibited concurrent skincare: Within 1 month after treatment, patients were forbidden to use retinoids (e.g., tretinoin), alpha‐hydroxy acids (e.g., glycolic acid), or other exfoliating products; daily application of broad‐spectrum sunscreen (SPF ≥ 30, PA+++) was required to prevent photoaging interference with outcomes.

### 2.5 Observation Index

#### 2.5.1 Primary and Secondary Endpoints

Primary endpoint: Fitzpatrick Wrinkle and Laxity Classification Scale (FWCS) score at 3 months post‐treatment (the core indicator for evaluating wrinkle/laxity improvement, as facial rejuvenation efficacy is typically judged by long‐term wrinkle reduction).

Secondary endpoints: (1) FWCS scores at 1 and 2 months post‐treatment; (2) VISIA‐derived skin texture and pore scores at 1, 2, and 3 months post‐treatment; (3) Global Aesthetic Improvement Scale (GAIS) score at 3 months post‐treatment; (4) Cutometer skin rebound rate at 1, 2, and 3 months post‐treatment; (5) Skin elasticity tester rebound rate (8 mm probe, 5‐s suction/3‐s release) at 1, 2, and 3 months post‐treatment; (6) Patient satisfaction rate at 3 months post‐treatment; (7) Incidence of adverse events within 1 week post‐treatment.

#### 2.5.2 Clinical Efficacy (FWCS Assessment)

The Fitzpatrick Wrinkle and Laxity Classification Scale (FWCS) [19] was used to evaluate wrinkle and laxity severity at baseline (1 day before treatment) and 1, 2, 3 months post‐treatment. The scale is defined as: Grade I (1–3 points): Shallow wrinkles, mild elastic tissue degeneration, subtle deepening of skin lines (no clinical laxity); Grade II (4–6 points): Moderate number of shallow‐to‐moderate wrinkles, moderate elastic tissue degeneration, obvious papular elastic tissue degeneration and pigment changes (mild laxity); Grade III (7–9 points): Large number of shallow‐to‐deep wrinkles, redundant skin folds (if present), severe elastic tissue degeneration, yellowish/pale skin with diamond‐shaped texture (moderate‐to‐severe laxity).

The minimal clinically important difference (MCID) of FWCS is 1 point (a decrease of ≥ 1 point indicates clinically meaningful improvement).

#### 2.5.3 Scoring of Skin Texture and Pore Status

The skin pore and texture status of the two groups was evaluated by the VISIA‐CR 7.0 digital skin analysis system (Canfield Scientific, Fairfield, NJ, USA) 1 day before treatment and 1, 2, and 3 months after the end of treatment. The indicator type was percentile scoring (based on a database of same‐age individuals, 0–100 points; higher scores indicate better skin texture/pore status than more peers). All assessments were performed by 2 blinded evaluators (unaware of patient grouping) who received pre‐assessment consistency training (Cohen's κ = 0.81, indicating good inter‐rater reliability).

#### 2.5.4 GAIS Score

The Global Aesthetic Improvement Scale (GAIS) was used to assess facial rejuvenation improvement at baseline (1 day before treatment) and 3 months post‐treatment. Two independent blinded evaluators scored facial rejuvenation improvement based on pre/post‐treatment photographs. The GAIS scale is defined as: 1 = very poor (significant worsening), 2 = poor (mild worsening), 3 = no change, 4 = improved (mild‐to‐moderate improvement), 5 = significantly improved (marked improvement) [21].

Inter‐rater reliability was pre‐evaluated using Cohen's kappa coefficient, with κ = 0.82 (95% CI: 0.65–0.99), indicating good consistency between the two evaluators. Discrepancies between scores were resolved by joint discussion until consensus was reached. Statistical analysis used the Wilcoxon rank‐sum test (non‐parametric) to compare groups, with results reported as median [interquartile range (IQR)] and proportion of patients with “improved/significantly improved” (GAIS ≥ 4).

#### 2.5.5 Skin Elasticity Test

Skin resilience was determined by standard negative pressure adsorption using a Cutometer MPA580 (Courage+Khazaka, Cologne, Germany). The device parameters were set as follows: 8 mm diameter, test mode R0 (elastic recovery), adsorption time 5 s, relaxation time 3 s. All patients were tested at the periocular area (1 cm from the outer canthus) 1 day before treatment and 1, 2, and 3 months after treatment; each test point was repeated 3 times to calculate the average value, and the skin rebound rate was recorded. The closer the value was to the state of healthy young skin (1.35–1.40 mm/s), the better the skin elasticity [22]. All tests were performed by blinded evaluators (unaware of patient grouping), and the device was calibrated daily using a standard calibration module to ensure data accuracy.

#### 2.5.6 Patient Satisfaction

Three months after the treatment, the patient's satisfaction was counted in the form of follow‐up and was categorized into three grades: very satisfied, satisfied, and dissatisfied. Total satisfaction rate = (very satisfied + satisfied) number of cases/total number of cases × 100%.

#### 2.5.7 Adverse Reactions

After treatment, observe the occurrence of adverse reactions (itching, pain, redness, temporary pigmentation, subcutaneous necrosis, etc.) in the two groups and count the incidence of adverse reactions. Incidence rate of adverse reactions = number of cases of adverse reactions/total number of cases × 100%.

### 2.6 Statistical Analysis

SPSS 22.0 software was used for analysis with the following detailed protocols: repeated measures (e.g., FWCS, Cutometer rebound rate at baseline/1/2/3 months) were analyzed via linear mixed‐effects models (LMM) with fixed effects of “group” (layered vs. conventional), “time” (baseline/1/2/3 months), and “group×time interaction” (to test whether group differences vary by time), and post hoc comparisons used Bonferroni correction to control Type I error; the Holm‐Bonferroni method was applied to adjust P‐values of secondary endpoints (e.g., FWCS at 1/2 months, skin texture scores) to control the family‐wise error rate (FWER ≤ 0.05); Shapiro–Wilk test was used for the normality of continuous data (e.g., FWCS, rebound rate) with all *p* > 0.05 confirming normality, and Levene's test was used for homoscedasticity with all *p* > 0.05 confirming equal variances; for count data, patient satisfaction (3‐level: very satisfied/satisfied/dissatisfied) was collapsed to binary (satisfied vs. not satisfied) and analyzed via Fisher's exact test (due to small cell counts, *n* = 1 in the layered group's “dissatisfied”), with the 95% confidence interval (CI) for the risk difference reported; for continuous outcomes (e.g., rebound rate), effect sizes were reported as “mean difference (MD) ± 95% CI” to reflect clinical relevance; statistical significance was defined as two‐tailed *p* < 0.05.

## 3 Results

### 3.1 Clinical Efficacy (FWCS Score)

**FIGURE 1:**
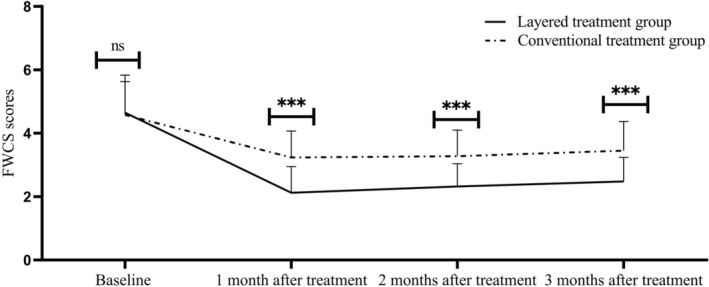
Trends in FWCS scores of patients in the two groups at different timepoints. Data are presented as mean ± standard deviation (SD). *p < 0.05, **p < 0.01, ***p < 0.001 versus conventional treatment group at the same timepoint; FWCS = Fitzpatrick Wrinkle and Laxity Classification Scale.

There was no significant difference in the baseline FWCS scores between the two groups (*p* = 0.865). At each time point after treatment, the FWCS scores in both groups showed an absolute decrease compared with the baseline, and the improvement in the stratified group was significantly greater: 1 month after treatment: the stratified group had an absolute decrease of 2.53 points, and the conventional group had a decrease of 1.34 points (*p* = 0.001); 2 months after treatment: the stratified group had a decrease of 2.33 points, and the conventional group had a decrease of 1.30 points (*p* = 0.002); 3 months after treatment: the stratified group had a decrease of 2.17 points, and the conventional group had a decrease of 1.13 points (*p* = 0.004). According to the minimal clinically important difference (MCID = 1 point [13]) of FWCS, the score decreases in both groups after treatment reached the MCID threshold, confirming that both groups had clinically significant wrinkle improvement. It is worth noting that the 3‐month decrease in the stratified group (2.17 points) exceeded the MCID by 1.17 points, while that in the conventional group only exceeded the MCID by 0.13 points—this means that the wrinkle reduction brought by the stratified technique was more significant, and patients could intuitively perceive the difference. The specific data are shown in Table 2. The trend of FWCS scores over time in the two groups is shown in Figure 1. The scores of the stratified group at each time point were significantly lower than those of the conventional group, and the downward trend was more stable (*p < 0.05).

**TABLE 2:** Comparison of FWCS scores between the two groups (*x* ± *s*, points).

Group	Baseline (before treatment)	1 month after treatment	2 months after treatment	3 months after treatment (primary endpoint)
Layered treatment group, *n* = 15	4.65 ± 1.18	2.12 ± 0.83	2.32 ± 0.72	2.48 ± 0.76
Conventional treatment group, *n* = 15	4.58 ± 1.05	3.24 ± 0.83	3.28 ± 0.82	3.45 ± 0.92
*t*	0.172	3.695	3.407	3.148
*p*	0.865	< 0.001^a^	0.002^a^	0.004
Within‐group change from baseline (points)	—	−2.53	−2.33	−2.17
Clinical significance (vs. MCID = 1 point)	—	Meets MCID	Meets MCID	Meets MCID

^a^ *p* value adjusted by the Holm–Bonferroni method (secondary endpoints); Primary endpoint: FWCS score at 3 months post‐treatment (no adjustment needed); MCID: Minimal clinically important difference (a decrease of ≥ 1 point indicates meaningful improvement); “Layered treatment group” replaces “Stratified treatment group” for terminology consistency.

### 3.2 Skin Texture and Pore State Scores

In the present study, no significant difference was observed between the two groups in terms of skin texture at 1 month after treatment; at 2 months after treatment, the two groups began to show significant differences, with the layered treatment group being superior to the conventional treatment group. At 3 months post‐treatment, both groups showed a decrease in skin texture scores, with the layered treatment group maintaining skin texture better than the conventional treatment group at 2 and 3 months postoperatively, and the difference was significant (*p* < 0.05). In terms of pores, only at 3 months postoperatively, there was a significant difference in the scores between the two groups (*p* < 0.05), and the layered treatment group had a better retention effect than the conventional treatment group. See Table 3.

**TABLE 3:** Comparison of skin texture and pore status scores between the two groups (*x* ± *s*, points).

Index	Group	Baseline	1 month after treatment	2 months after treatment	3 months after treatment
Skin texture score	Layered treatment group, *n* = 15	68.52 ± 6.34	62.38 ± 6.18	58.42 ± 6.23	42.25 ± 5.72
	Conventional treatment group, *n* = 15	67.86 ± 6.42	61.68 ± 6.24	50.26 ± 6.56^a^	36.56 ± 4.82^a^
	*p* (between groups)	0.721	0.815	0.023	0.018
Pore score	Layered treatment group, *n* = 15	67.34 ± 6.51	61.25 ± 6.32	59.12 ± 6.48	41.56 ± 6.82
	Conventional treatment group, *n* = 15	66.82 ± 6.63	60.28 ± 5.88	56.34 ± 6.25	34.28 ± 5.16^a^
	*p* (between groups)	0.835	0.792	0.186	0.021

^a^ *p* < 0.05 versus layered treatment group; Higher scores indicate better skin condition; Baseline *p* > 0.05 indicates no significant difference between groups before treatment; VISIA version 7.0 was used for assessment.

### 3.3 GAIS Score

As assessed by 2 blinded independent evaluators, the layered treatment group had a significantly higher median GAIS score than the conventional group (4 [interquartile range, IQR: 4–5] vs. 3 [IQR: 3–4], *Z* = 3.862, *p* < 0.001). The proportion of patients with “improved” or “significantly improved” (GAIS score ≥ 4) was 100.0% (15/15) in the layered group, compared with 66.7% (10/15) in the conventional group, as shown in Table 4.

**TABLE 4:** Comparison of GAIS scores between the two groups of patients.

Group	Median [IQR] (GAIS score, points)	*Z* value	*p* value	Proportion of patients with GAIS ≥ 4 (%)
Layered treatment group, *n* = 15	4 [4–5]	3.862	< 0.001	100.0 (15/15)
Conventional treatment group, *n* = 15	3 [3–4]	—	—	66.7 (10/15)

*Note: GAIS* = Global Aesthetic Improvement Scale (1 = very poor, 2 = poor, 3 = no change, 4 = improved, 5 = significantly improved); Statistical analysis using Wilcoxon rank‐sum test (*Z* test); IQR = interquartile range; GAIS ≥ 4 indicates “improved” or “significantly improved”.

### 3.4 Skin Elasticity Test

**FIGURE 2:**
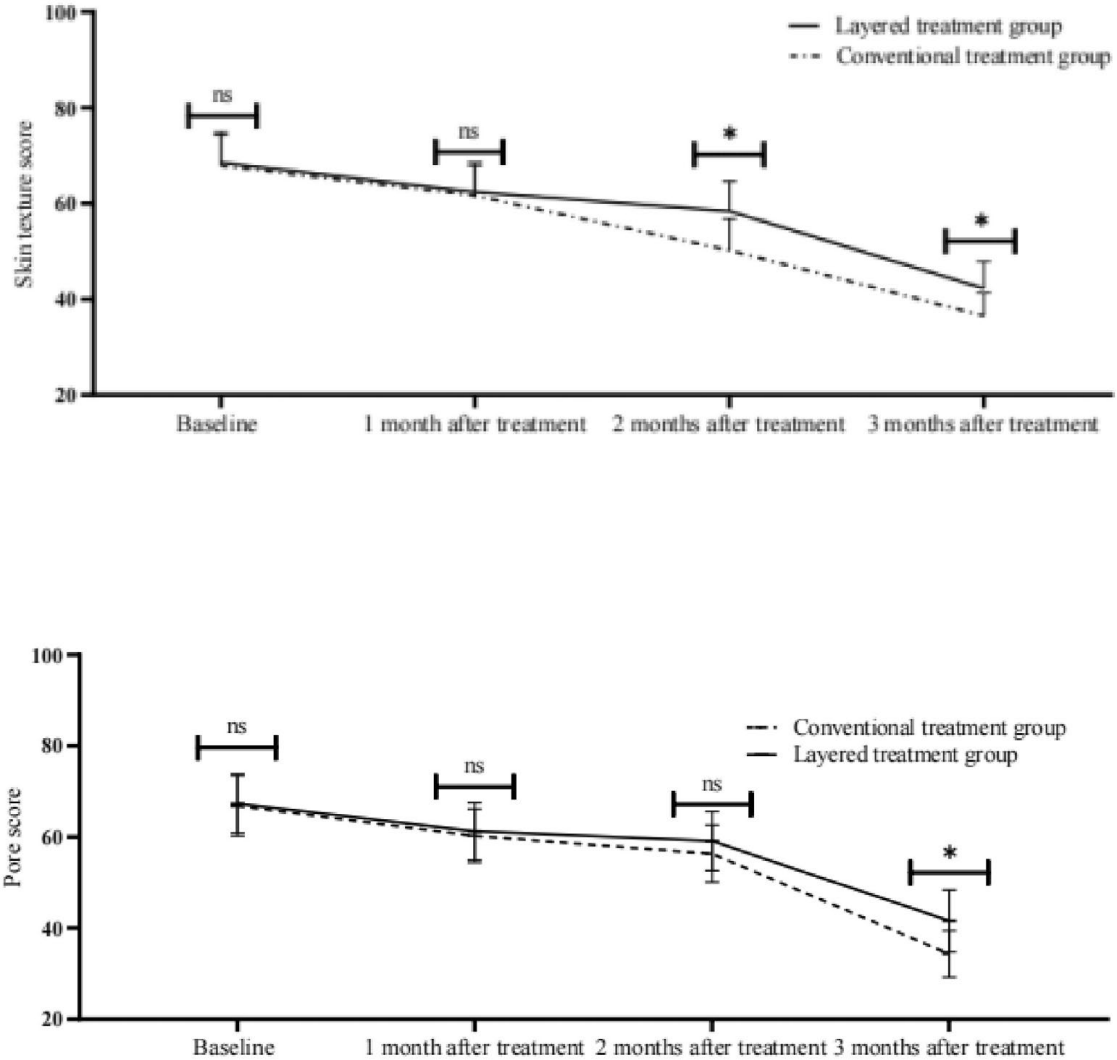
Trends in skin rebound rate of patients in the two groups at different timepoints. Data are presented as mean ± standard deviation (SD). *p < 0.05, **p < 0.01, ***p < 0.001 versus conventional treatment group at the same timepoint; rebound rate was measured by Cutometer MPA580 (2 mm diameter probe, 450 mbar suction pressure).

The skin resilience rate was similar in both groups before treatment, with no significant difference (*p* > 0.05). At 1 month after treatment, the rebound rate of both groups showed an increasing trend with no significant difference (*p* > 0.05). At 2 and 3 months post‐treatment, the rebound rate of the layered treatment group was significantly higher than that of the conventional treatment group, with statistically significant differences (both *p* < 0.001), Table 5. The time trend of skin rebound rate in the two groups is shown in Figure 2. The rebound rate of the layered group was significantly higher than that of the conventional group from 2 months onwards, and at 3 months, it was closer to the level of healthy young skin (**p < 0.01).

**TABLE 5:** Comparison of skin rebound rate between the two groups x±s,mm/s.

Group	Before treatment	1 month after treatment	2 months after treatment	3 months after treatment	Group×time interaction (LMM)
Layered treatment group, *n* = 15	0.82 ± 0.11	1.00 ± 0.12	1.20 ± 0.13	1.34 ± 0.10	*F* = 12.65, *p* < 0.001
Conventional treatment group, *n* = 15	0.83 ± 0.10	0.92 ± 0.10	1.00 ± 0.12	1.10 ± 0.11	—
*t* (between groups)	0.261	1.984	4.378	6.253	—
*p* (between groups)	0.796	0.057	< 0.001	< 0.001	—
MD (95% CI) versus conventional group	−0.01 (–0.08 to 0.06)	0.08 (–0.01 to 0.17)	0.20 (0.11 to 0.29)	0.24 (0.16 to 0.32)	—

*Note:* LMM = Linear mixed‐effects model; MD = Mean difference; Cutometer protocol: MPA 580 probe (2 mm diameter), suction pressure 450 mbar, suction time 2 s, relaxation time 2 s; “Layered treatment group” replaces “Stratified treatment group” for terminology consistency.

### 3.5 Patient Satisfaction

In the present study, the satisfaction of the Layered treatment group was significantly higher than that of the conventional treatment group (93.33% vs. 60.00%), and there was a significant difference (*p* < 0.05), as shown in Table 6.

**TABLE 6:** Comparison of patient satisfaction between the two groups.

Group	Very satisfied (*n*, %)	Satisfied (*n*, %)	Dissatisfied (*n*, %)	Satisfaction rate (*n*, %)	Fisher's exact test (*p* value)	95% CI for risk difference (layered vs. conventional)
Layered treatment group, *n* = 15	13 (86.67)	1 (6.67)	1 (6.67)	14 (93.33)	0.031	13.33%–53.33%
Conventional treatment group, *n* = 15	8 (53.33)	1 (6.67)	6 (40.00)	9 (60.00)	—	—

*Note:* Satisfaction rate = (Very satisfied + Satisfied)/Total cases × 100%; Risk difference = Satisfaction rate of layered group—Satisfaction rate of conventional group; “Layered treatment group” replaces “Stratified treatment group” for terminology consistency; χ^2^ test replaced with Fisher's exact test due to small cell counts (*n* = 1 in layered group's “Dissatisfied”).

### 3.6 Adverse Reactions (AEs)

In the present study, the skin of the conventional treatment group showed visible flushing after treatment, and returned to normal 1–3 days after the end of treatment, and the skin of the layered treatment group showed visible redness and swelling, and returned to normal 3–5 days after the end of treatment. No adverse reactions such as hyperpigmentation, skin sensitivity, wound infection, etc. were observed in both groups, as shown in Table 7.

**TABLE 7:** Detailed adverse events in the two groups.

Adverse event type	Group	Incidence rate (*n*, %)	Severity (mild/moderate/severe)	Mean duration (± SD, days)	Remission method
Erythema	Layered treatment group, *n* = 15	15 (100%)	15/0/0	3.2 ± 0.8	Spontaneous resolution (no medication needed)
	Conventional treatment group, *n* = 15	15 (100%)	15/0/0	1.8 ± 0.6	Spontaneous resolution
Edema	Layered treatment group, *n* = 15	15 (100%)	15/0/0	3.5 ± 0.9	Spontaneous resolution
	Conventional treatment group, *n* = 15	0 (0%)	—	—	—
Pruritus	Layered treatment group, *n* = 15	2 (13.33%)	2/0/0	2.1 ± 0.5	Relieved by cold compress
	Conventional treatment group, *n* = 15	1 (6.67%)	1/0/0	1.9 ± 0.4	Relieved by cold compress

*Note:* Definition of severity: Mild = does not affect daily life; Moderate = slightly affects daily life; Severe = significantly affects daily life. The duration of AEs in the stratified group (erythema: 3.2 ± 0.8 days, edema: 3.5 ± 0.9 days) was longer than that in the conventional group, with a corresponding longer downtime (3–5 days vs. 1–3 days).

### 3.7 Typical Cases

**FIGURE 3:**
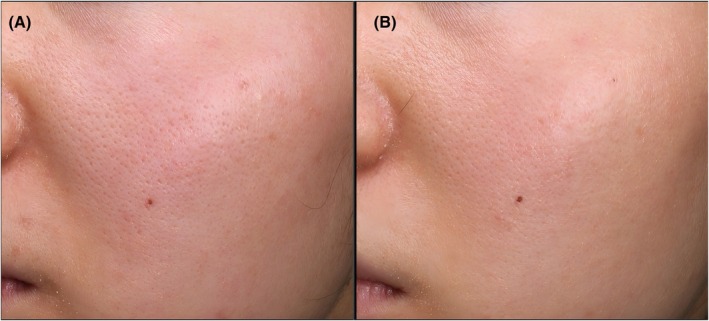
Typical case 1 (female, 43 years old). (A) Obvious enlarged pores pre‐treatment; (B) Significant pore shrinkage 1 month post‐treatment with layered HFM.

Case 1 (female, 43 years old): Pre‐treatment (Figure 3A): Obvious enlarged pores (VISIA pore score = 67.34) and dull skin; 1 month post‐treatment (Figure 3B): Significant pore shrinkage (VISIA pore score = 61.25), confirmed by 2 blinded evaluators (GAIS score = 5, “much improved”).

**FIGURE 4:**
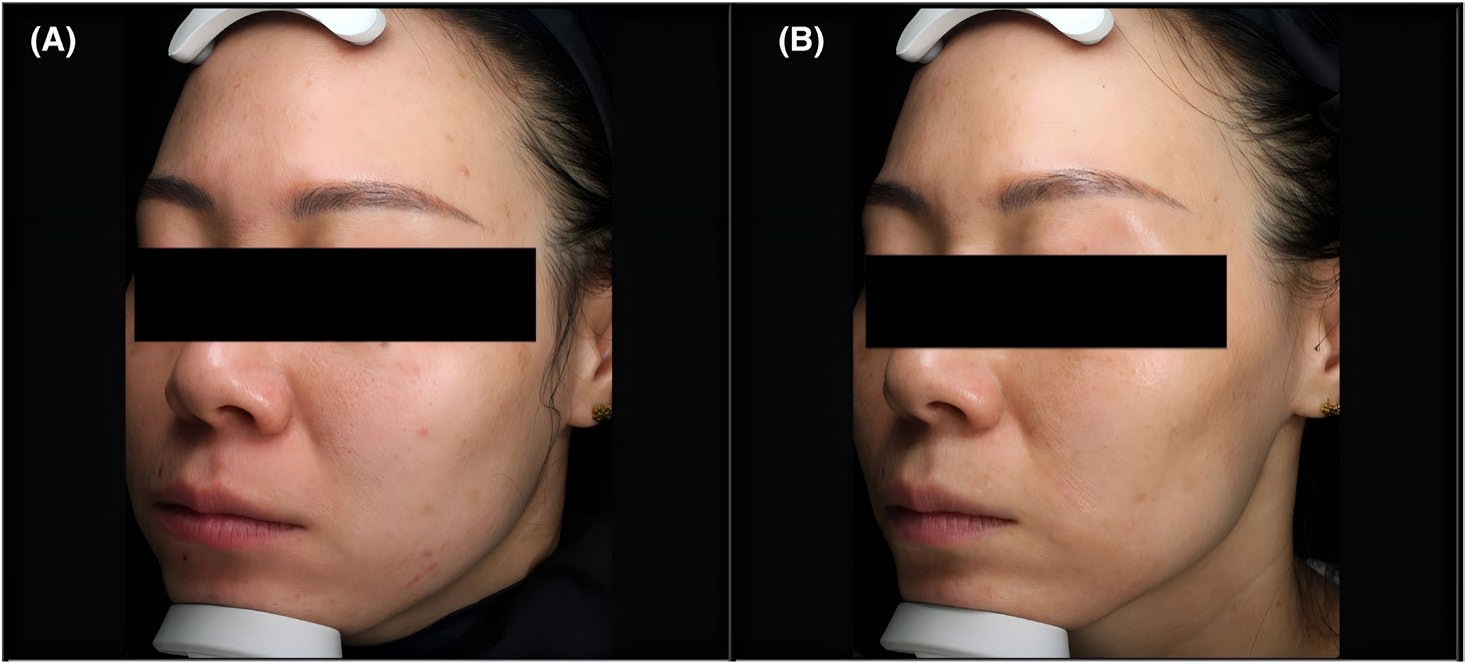
Typical case 2 (female, 49 years old). (A) Obvious skin laxity pre‐treatment; (B) Significant improvement in skin laxity 1 month post‐treatment with layered HFM.

Case 2 (female, 49 years old): Pre‐treatment (Figure 4A): Obvious skin laxity (FWCS score = 5) and dull complexion; 1 month post‐treatment (Figure 4B): Marked improvement in laxity (FWCS score = 2), confirmed by 2 blinded evaluators (GAIS score = 4, “improved”).

Photography conditions consistent with Methods 1.4; no post‐processing beyond exposure/white balance adjustment.

## 4 Discussion

The phenomenon of facial aging encompasses multiple levels of degeneration of the skin, superficial fat, superficial musculotendinous system, ligaments and intervals, deep fat, periosteum, and deep fascia to bone. It is mainly characterized by age‐related facial defects and aging features, accompanied by multiple areas of laxity and sagging [20, 23]. Given the broad and flat nature of the Chinese facial structure, its unique physical characteristics, and aesthetic standards, the facial aging process differs from that of Westerners [24, 25]. Therefore, targeted aesthetic treatment strategies are needed to improve the flat appearance of specific areas and to restore the signs of aging triggered by decreased tissue volume and sagging. High‐frequency microneedling layering technology enables finer stimulation of the skin layers by targeting needle length, power, and treatment parameters at different skin levels, resulting in more precise treatment results [26, 27]. The aim of this study was to evaluate the clinical efficacy, skin quality improvement, and patient satisfaction of the high‐frequency microneedle layering technique in facial rejuvenation treatment, in order to provide a more optimal treatment plan for solving facial aging problems.

The results of this study showed that both the standard HF microneedling technique and the HF microneedling layering technique demonstrated positive results in improving facial skin conditions, but the HF microneedling layering technique was superior to conventional treatments in several aspects. First, in terms of clinical efficacy, this study assessed changes in facial wrinkles and laxity in patients before and after treatment by FWCS. The results showed that although there was no significant difference in the FWCS scores of the two groups of patients before treatment, the FWCS scores of the layered treatment group decreased significantly more than those of the conventional treatment group at 1 month after treatment, and the scores of the layered treatment group were consistently lower than those of the traditional treatment group at 2 and 3 months after treatment. This suggests that the high‐frequency microneedle layering technique is more effective in improving facial wrinkles and laxity in both the short and long term. The layered treatment precisely modulates the thermal effect through different levels of microneedle depth, which may have promoted the production of collagen and elastin fibers at a deeper level; however, this mechanistic inference lacks direct histologic evidence (e.g., dermal collagen staining) or imaging verification (e.g., high‐frequency ultrasound) in the present study, and needs to be confirmed by subsequent studies incorporating pathological assessment [16]. In the assessment of skin texture and pore status, the layered treatment group also showed significantly better improvement than the conventional treatment group. This suggests that the high‐frequency microneedle layering technique can provide longer‐lasting skin improvement effects, especially in delaying aging and improving pore size, which may be attributed to the fact that microneedles at different depths combined with the thermal effect of radiofrequency can better stimulate the deeper structure of the skin and promote the regeneration of elastic fibers and the remodeling of collagen; yet similar to the aforementioned mechanism, this hypothesis remains unvalidated by our study's data and requires further histopathological confirmation. This layered treatment strategy makes the treatment effect not only more significant but also lasts longer.

The GAIS score, one of the criteria for evaluating facial rejuvenation effect, was significantly higher in the layered treatment group than in the conventional treatment group in this study, further supporting the advantages of layered treatment in facial rejuvenation effect. By precisely regulating the facial skin, layered treatment can provide more uniform and long‐lasting improvement and help patients achieve a more natural facial rejuvenation effect. In addition, skin elasticity improvement was significantly and positively correlated with decreased FWCS scores and increased GAIS scores, confirming that elasticity restoration is an important phenotype of facial rejuvenation. Taking the median healthy young skin resilience rate (1.375 mm/s) as a benchmark, the resilience rate of the layered treatment group approached 97% of the healthy young skin range at 3 months, which was significantly higher than that of the conventional group (80%), a difference that may be attributed to the fact that single‐layer microneedling stimulates only localized vertical collagen proliferation, whereas layered technology forms a three‐dimensional mesh restorative scaffold through multi‐depth thermal injury, which is closer to the physiological elasticity mesh arrangement structure of fibers [28]. Again, this structural inference lacks direct evidence from our study and should be interpreted with caution pending verification by tissue section observations.

Patient satisfaction is an important dimension in assessing treatment efficacy, and patient satisfaction was significantly higher in the layered treatment group than in the conventional treatment group in this study. This difference was closely related to the significant difference in treatment effect, and the higher treatment effect in the Layered treatment group directly translated into higher patient satisfaction. In addition, patients' satisfaction with the improvement in appearance and the durability of the effect after treatment further reflects the advantages of the layered treatment technique in actual clinical application.

### 4.1 Efficacy and Downtime Trade‐Off

In terms of adverse reactions, no serious adverse events were observed in either group, but there were notable differences in transient reactions and corresponding downtime (Table 7). In the conventional treatment group, only skin flushing occurred post‐treatment, with a mean duration of 1.8 ± 0.6 days, corresponding to a downtime of 1–3 days. In contrast, the layered treatment group experienced both mild erythema (mean duration 3.2 ± 0.8 days) and edema (mean duration 3.5 ± 0.9 days), resulting in a longer downtime of 3–5 days. This difference in recovery period is consistent with the consensus that deeper and multi‐layered dermal stimulation correlates with prolonged inflammatory response and recovery time in microneedle therapy [7, 20]. Clinically, this trade‐off requires full communication with patients before treatment: for individuals with high social activity demands, the shorter downtime of conventional treatment may be more acceptable, while patients prioritizing long‐lasting and significant efficacy (e.g., for moderate‐to‐severe wrinkles) are more willing to tolerate the extended recovery period of layered treatment—a preference reflected in the higher satisfaction rate (93.33% vs. 60.00%) of the layered group. Such personalized assessment of efficacy‐demand and downtime‐tolerance is critical for optimizing clinical decision‐making [15, 29].

### 4.2 Limitations

However, there are still some limitations in this study. First, the sample size was small, covering only 30 patients, so extrapolation of the findings may be limited. Second, despite the obvious treatment effect, this study only assessed the maintenance of the treatment effect in the short term, and future studies should expand the sample size and conduct long‐term follow‐up to assess the long‐term maintenance of the treatment effect. Third, the mechanistic inferences proposed in this discussion lack direct histologic support, and subsequent studies should incorporate dermal tissue sampling and molecular detection (e.g., collagen type I/III expression) to verify the biological basis of layered treatment advantages. In addition, the effects of different microneedle parameters (e.g., needle length, power, and pulse width) on the treatment effect of stratified therapy are unclear, and the specific mechanisms of the effects of different treatment parameters on clinical efficacy can be explored in the future.

## 5 Conclusion

In conclusion, high‐frequency microneedle layering technology has obvious advantages in facial rejuvenation treatment compared with conventional treatment, which can effectively improve facial skin texture, wrinkles, pore status, and skin elasticity, and provide longer‐lasting improvement effects. Meanwhile, layered treatments have significantly higher patient satisfaction than conventional treatments and are safe with only mild, transient adverse effects—though at the cost of slightly longer downtime. Future studies should further explore the long‐term maintenance of the treatment effect, optimize the best treatment parameters, and validate the underlying mechanisms through histopathological evidence.

## Author's Contribution

Ye Tang and Kejia Wu designed the research study; Ye Tang, Kejia Wu, Xin Song, Zhishang Lu, Meina Fan, and Manli Qiao performed the research; Ye Tang and Kejia Wu collected and analyzed the data. Xin Song and Zhishang Lu have been involved in drafting the manuscript, and all authors have been involved in revising it critically for important intellectual content. All authors give final approval of the version to be published. All authors have participated sufficiently in the work to take public responsibility for appropriate portions of the content and agreed to be accountable for all aspects of the work in ensuring that questions related to its accuracy or integrity.

## Funding

The authors have nothing to report.

## Ethics Statement

This research project has been approved by the Ethics Committee of Changzhou Third People's Hospital and operated in strict accordance with ethical standards. In this study, we respect and protect the rights and privacy of participants and ensure the confidentiality of their personal information. (1) Participants' informed consent: We explained the purpose, process, risks and benefits of the study to all individuals involved in the study orally or in writing, and obtained their informed consent. Participants have the right to know that their participation is voluntary and can withdraw from the study at any time. (2) Data confidentiality and privacy protection: We have taken appropriate measures to protect the privacy of participants' personal information. We will not disclose any personal information that may lead to the identification of participants. In the research report, we will treat the participants' information anonymously. (3) Assessment and management of potential risks: We assessed the potential risks that may be involved in the study during the project design stage and took appropriate measures to reduce or manage these risks. We guarantee that participants will not suffer any physical or psychological harm because of participating in the study. (4) Research data use: We will strictly abide by the principles of legality and transparency in data use to ensure the correct use and interpretation of research data. We will try our best to avoid data misunderstanding and abuse, and only use the data for research purposes The purpose of this statement is to ensure the ethical compliance of research projects and protect the rights and privacy of participants. If you have any further questions or doubts, please feel free to contact us.

## Conflicts of Interest

The authors declare no conflicts of interest.

## Supporting information


**Figure S1:** Patient flow diagram.

**Table S1:** Baseline characteristics of patients in the two groups.

## Data Availability

The raw data in this study are not publicly available due to privacy restrictions of patient clinical information. However, de‐identified data can be obtained from the corresponding author upon reasonable request.
