# Supplementary material for: Comparative Evaluation of High‐Frequency Microneedling Using a Layering Technique Versus Conventional Technique for Facial Rejuvenation
Source: J Cosmet Dermatol. 2026 Feb 25;25(3):e70651. doi: 10.1111/jocd.70651 (PMC12936247; doi:10.1111/jocd.70651)
Supplement: Supplementary file 2 — Table S1: Baseline characteristics of patients in the two groups. [file JOCD-25-e70651-s001.docx]

Table S1 Baseline characteristics of patients in the two groups

| Characteristic | Layered treatment group $n=15$ | Conventional treatment group $n=15$ | p |
| --- | --- | --- | --- |
| Age (years,x±s) | 37.18±6.22 | 38.25±5.88 | 0.723 |
| Gender (n,%) |  |  | 0.891 |
| - Female | 13 (86.67) | 12 (80.00) |  |
| - Male | 2 (13.33) | 3 (20.00) |  |
| Fitzpatrick skin type (n,%) |  |  | 0.915 |
| - Type III | 9 (60.00) | 8 (53.33) |  |
| - Type IV | 6 (40.00) | 7 (46.67) |  |
| Baseline FWCS score (points,x±s) | 4.65±1.18 | 4.58±1.05 | 0.865 |
| Baseline VISIA texture score (points,x±s) | 68.52±6.34 | 67.86±6.42 | 0.721 |
| Baseline VISIA pore score (points,x±s) | 67.34±6.51 | 66.82±6.63 | 0.835 |
| Baseline skin rebound rate $mm/s,x\pm s$ | 0.82±0.11 | 0.83±0.10 | 0.796 |
| Prior facial treatment history (n,%) | 0 (0.00) | 0 (0.00) | 1.000 |
| Smoking status (n,%) | 2 (13.33) | 2 (13.33) | 1.000 |
| Note:FWCS = Fitzpatrick Wrinkle and Laxity Classification Scale;P>0.05 indicates no significant difference between groups at baseline. | | | |
